# Effect of Limestone and Quartz Fillers in UHPC with Calcined Clay

**DOI:** 10.3390/ma15217711

**Published:** 2022-11-02

**Authors:** Guillermo Hernández-Carrillo, Alejandro Durán-Herrera, Arezki Tagnit-Hamou

**Affiliations:** 1 Universidad Autónoma de Nuevo León, Facultad de Ingeniería Civil, Ciudad Universitaria, Ave. Universitaria S/n, San Nicolás de los Garza 66455, NL, Mexico; 2 Faculté de Genié, Université de Sherbrooke, 2500 Boulevard de l’Université, Sherbrooke, QC J1K 2R1, Canada

**Keywords:** ultra-high-performance concrete, metakaolin, limestone filler, C-A-S-H, monocarboaluminate

## Abstract

Ultra-high-performance concrete (UHPC) is a material developed to maximize the engineering characteristics of hydraulic concrete, in terms of durability and mechanical properties, but the adoption of this technology in practice has not turned out as desired, mainly due to the high amounts of cement and silica fume required for its production, and for its consequences on both economic and ecological costs. As an option to improve the impact of UHPC, both on costs and on sustainability, this work evaluates four UHPC series with metakaolin additions of 5%, 10%, 15% and 20%, and the substitution of 37.5% of the Portland cement volume by limestone or quartz filler. The compressive strength, the bulk electrical resistivity and a set of tests for microstructural characterization (TGA, XRD and quantitative EDS) were utilized to better understand the role of calcite on the hydration and pozzolanic reactions in ternary Portland cement-metakaolin-limestone filler. Results indicate that the reaction of calcite is scarce and should be considered as a mere filler, as no increase in AFm phases were found. Nevertheless, the ternary mixture with 15% of metakaolin in addition to cement, and with 37.5% of the Portland cement volume substituted by limestone filler, was the one that presented the best performance in terms of compressive strength and bulk electrical resistivity. The results of the microstructural characterization indicate that the high kaolin content in the metakaolin originated the most significant hydration and pozzolanic reactions development between the ages of 7 and 28 days, as between 28 and 91 the reaction remained dormant. In general, the whole set of results included in this work indicate that limestone filler doesn’t act as a better filler than other kind of powders when used in ternary Portland cement-metakaolin- filler systems.

## 1. Introduction

The ultra-high-performance concrete (UHPC) was conceived with the idea to take Portland cement concrete to its limits in terms of durability and mechanical performance. Nevertheless, the high amounts of cement (usually more than 900 kg/m^3^) [1,2] and silica fume [3] required to produce it, have been a barrier to faster transfer into practice because of its high cost and high ecological impact.

In contrast, remarkable benefits of UHPC that counteract the negative impact on both economic and ecological costs are associated with its ultra-high mechanical properties and durability; first because for a specific application and in relation to a conventional concrete, in structural design, these characteristics will lead to structural elements of smaller dimensions that will require smaller concrete volume for their construction; and second, because its superior durability will increase the potential service life of structures that in service will be subject to environmental conditions that typically cause the deterioration of concrete elements built with conventional concrete.

Nowadays, the production of Portland cement contributes 10% of anthropogenic emissions [4], although this contribution could be considered constant, due to the increase in the demand for concrete that is expected in the immediate future (especially in the developing countries), the net contribution (in terms of tons of CO_2_/ton of cement) coming from the cement industry, will be increased proportionally to the consumption of this material. However, this negative impact on sustainability can also be counteracted through a greater use of UHPC in conjunction with limestone powder and calcined clays in concrete construction practice, since the use of limestone powder and calcined clays can reduce the environmental impact by 37.1%, compared to conventional UHPC in the practice of concrete construction; likewise, an appropriate limestone powder content can contribute to higher strength, denser pore structure, lower total free shrinkage, and higher sustainability efficiency [2,5].

It is proposed that UHPC mixes could make more durable structures while decreasing the volume of concrete due to their higher performance. As commonly demonstrated, the ratio of water to cement in UHPC is extremely low, and the extent of hydrated cement is very limited (30–40%) [6], which implies that plenty of unhydrated cement is only used as a filler. In order to alleviate the concerns of sustainability of the UHPC a ternary cementitious system of cement (HS)-limestone (L)-calcined clay or metakaolin (MK) has been proposed as an alternative in UHPC designs with success, resulting advantageous because the more costly unreacted cement is being replaced by limestone filler, in this regard, the limestone replacement for cement in low w/cm concretes appears to be one viable, but currently underutilized, option [7].

The availability of supplemental cementitious materials, such as slag and fly ash, depends on location, quantity, and the current market situation. However, clays are available in needed quantities around the world, and in view of the significant increase in cement demand and the need to reduce the clinker factor, calcined clays are becoming increasingly important to the cement industry as a material. Supplemental cement [8].

In order to search for an alternative to reduce the negative impacts in sustainability, attributable to the high consumption of cement and silica fume demanded by the UHPC, it has been reported that metakaolin could be an appropriate alternative to silica fume in UHPC [9], in this work, four ternary cementitious combinations of Portland cement and metakaolin were produced to evaluate the effect of the metakaolin as a supplementary cementitious material and matrix densification enhancer in UHPC, in combination with a limestone or quartz powder dosed in substitution of a fixed volume of the cement.

For cementitious systems like the ones studied in this work, it has been reported that the formation of carboaluminates resulting from the reaction of limestone, calcined clays and Portland cement can be the source that leads to the improvement of concrete properties [10]. However, it is expected that the low solubility of calcite and the low availability of water in a UHPC can interfere with the formation of carboaluminates. Therefore, the aim of this study was to define the dosage of metakaolin (5%, 10%, 15% and 20%) that presented the best performance at a macro level, in terms of compressive strength and bulk electrical resistivity at the ages of 7, 28 and 91 days, and for this optimized mixture to evaluate the influence of either the solubility of the calcite and/or its filler effect, at a micro level in the microstructural development, the hydration process, the formation of carboaluminates, and the Al/Ca ratio of the C-A-S-H phase, trough the following analytical techniques; thermogravimetry (TGA), X-ray diffraction (XRD) and Quantitative Energy Dispersive Spectroscopy (QEDS), respectively.

## 2. Experimental Investigation

### 2.1. Materials

The materials used in the research were as follows:

- An ASTM C 1157 Type HS cement (HS), with a Blaine fineness of 438 m^2^/kg, a mean particle size of 14 µm (0.4–80.0 µm), and a density of 3.18 g/cm^3^.
- An ASTM C 618 Metakaolin (MK), with a BET-specific surface area of 10.66 m^2^/g, a mean particle size of 14 µm (0.4–70.0 µm), and a density of 2.59 g/cm^3^.
- An ASTM C 994 Type A polycarboxylate based high range water reducer or superplasticizer (HRWRA), with a density of 1.09 g/cm^3^ and an active agent content of 40%.
- Quartz Powder (Q) with a mean particle size of 19 µm (2.0–80.0 µm), and a density of 2.75 g/cm^3^.
- Limestone Powder (L) with a mean particle size of 17 µm (0.6–400.0 µm), and a density of 2.71 g/cm^3^.
- An ASTM C 33 natural river sand with 5 mm maximum size (80.0 µm–5.0 mm), a density of 2.61 g/cm^3^ and an absorption of 1.15%.

**Figure 1:**
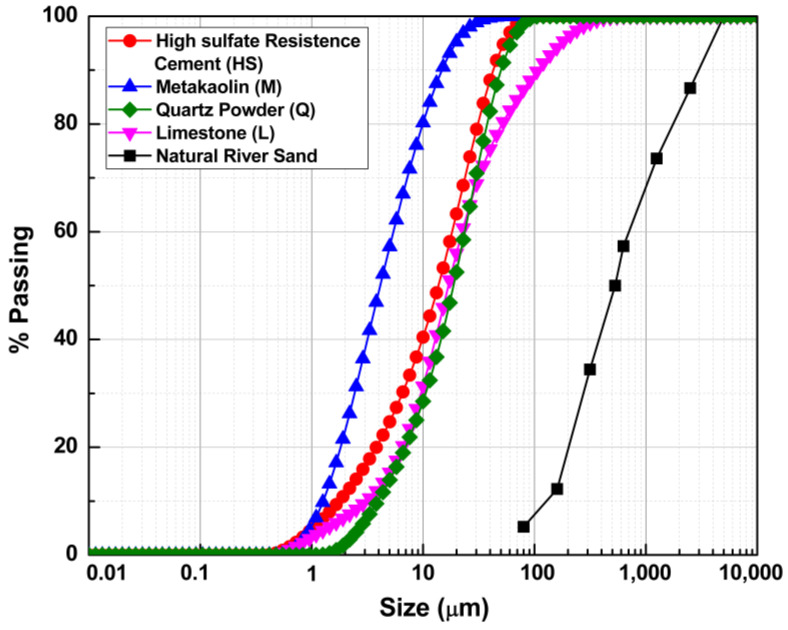
Particle size distribution of HS, MK, Q, L and the natural river sand.

Figure 1 presents the particle size distribution of cement metakaolin, limestone powder, quartz powder, and natural river sand. Table 1 summarizes chemical and physical properties for HS, MK, Q and LS. With the exception of the natural river sand and the quartz powder that is 100% quartz, Table 2 summarizes the normalized chemical phase composition for HS, MK and L.

### 2.2. Mixture Proportions

Power’s model is a simple approach for estimating the relative volumes of hydration products, porosity, and chemical shrinkage present in Portland cement paste as a function of its starting water-to-cement ratio (*w*/*c*) and current degree of hydration. It forms an important link between cement composition, microstructure, and performance, necessary for modeling cement-based systems [11]. Through this model, the estimated *w*/*c* to maximize cement hydration is around 0.4 [12].

For this research work, four base mix designs with a *w*/*c* of 0.25, a cement consumption of 800 kg/m^3^, a target flow of 250 ± 50 mm, and metakaolin additions of 5%, 10%, 15% and 20% were used to evaluate the reaction of limestone powder in a binary Portland cement-metakaolin UHPC cementitious system, and to confirm if the reaction of the limestone powder have the potential to detonate improvements in concrete performance. For this purpose and for each of the four base mix designs, two additional mixtures were produced, in which the cement volume was replaced by 37.5% of either limestone or quartz powder, and at the same time the cement content was decreased to 500 kg/m^3^ in order to reach a *w*/*c* of 0.40 for a fixed water dosage of 200 kg/m^3^.

To reach the target flow of 250 ± 50 mm, measured through the standard cone described in ASTM C 230 [13], a SP-HRWRA polycarboxylate based admixture was used. Nevertheless, the mixtures with an addition of 20% MK did not reach such level of fluidification without a slight vibration.

The proportions for the four series of mixtures evaluated in this work, as well as some fresh stage properties determined by duplicate are reported in Table 3, where in the nomenclature used to distinguish the different mixtures, the number indicates the percentage of metakaolin dosed in addition to the cement content (5, 10, 25 and 20%), and the letter at the end of some nomenclatures indicates the volume of cement substituted by limestone (L) or quartz (Q) powder. The amounts of metakaolin selected are those commonly used as partial substitution, such as those used by Alexander & Bakera [14]. In all the nomenclatures, M indicates metakaolin. As an example, mixture M15 is a mixture with 15% of metakaolin in addition to the cement mass, while the mixture M15L has the same amount of metakaolin but additionally a volume substitution of 37.5% of the cement by limestone powder.

The reductions of unit weight reported in Table 3 for mixtures with L or Q, are attributed to the differences between the density of the cement (3.18) and the limestone (2.75) or quartz powders (2.71). In this Table, the results of air content indicate that the compaction by vibration could eliminate the entrapped air by extracting from concrete between 10 and 30 L of air; this level of compaction also influences the unit weight and exhibits a very dense cementitious matrix.

To mix the ingredients and produce the twelve mixtures evaluated in this work, a 19-litre planetary mixer was used to make individual concrete batches of 2.5 L through the mixing procedure described below.

1. Approximately ninety percent of the water volume and all the other ingredients were added to the bowl.
2. The initial mixing consisted of 4 min at 104 rpm, during this time is believed that the aggregates play a role at breaking powders conglomerates.
3. After the initial mixing, the mixer was turned off for a period of one minute. The SP-HRWRA was mixed in the remaining volume of water to facilitate its homogenization with the other ingredients when pouring it into the mixing bowl at the end of the rest period. The delayed dosage of the admixture avoids the entrapment of the polycarboxylate chains within the first cement hydration products and improves its performance [15].
4. From minute sixth to tenth, the speed of the mixer was shifted to 194 rpm.
5. Finally, mixing continues at a speed of 353 rpm for 5 min, for a total mixing time of 15 min.

### 2.3. Test Methods

For the chemical and physical characterization of the powdered materials, the following analytical techniques and equipment’s were used:

- Elemental chemical composition—PANalytical X-ray Fluorescence Sprectometer, model Axios.
- Particle Size Distribution—Malvern Mastersizer 2000.
- Density—Quantachrome, Multi Pycnometer.
- Blaine Fineness—Slimatic by Interlab.
- BET specific surface area—Micromeritics ASAP 2020 Plus.
- Thermogravimetric analyses (TGA) were performed using TA instruments SDT Q600, on paste fractions reproduced from the proportions of mixtures that presented similar compressive strengths at 91 days, M15, M15L and M15Q. For this purpose, the pastes were pulverized by grinding, until the whole powder passes the sieve with a nominal opening of 150.0 µm (No. 100), and subsequently the solvent exchange method with isopropanol and diethyl ether was used to freeze the hydration process. The tests were carried out on 50 mg samples with a ramp of 10 °C/min within a temperature range of 25 to 1000 °C and a nitrogen flow of 50 mL/min. The data is processed using the universal analysis software TA 2000.
- TGA investigations allowed the determination of bound water and the mass loss corresponding to the portlandite decomposition. The principle is based on the measurement on the change of mass of the sample as a function of time and temperature.

In order to identify the crystalline phases and the amorphous content of the powders, XRD patterns were acquired with a PixCel1D detector form Bruker under a continuous scan from 5 to 70 2θ. Rietveld refinement of the anhydrous powders was performed according to the recommendations and procedures described by R. Snellings et al. and K. Scrivener et al. [16,17]. The amount of each phase was normalized through the external standard method, using for this purpose a corundum sample with known crystallinity of 98.2%, that was calibrated with the NIST’s corundum SRM 676a standard. Results are summarized in the Table 4.

For each mixture reported in Table 3, compressive strength was determined according to ASTM C 109, for which three (3 specimens) were casted, cured in a standard curing room (T = 23 ± 2 °C, RH ≥ 95 %, not immersed in water) until the scheduled testing age, and tested at the ages of 7, 28 and 91 days. At the same ages, these specimens were also used to determine the bulk electrical resistivity (BER), according to the test procedure described in ASTM C 1876 and using for this purpose the equipment Giatec RCON 2 with a frequency 1 KHz.

To identify the most sensitive phases to the reactions due to the interaction of the different species present in the study mixtures, and to quantify or qualify their concentrations, thermogravimetric an X-ray diffraction (XRD) analysis were performed in the paste fraction of the mixtures that presented similar compressive strengths at the age of 91 days. The thermogravimetric analysis (TGA) was conducted using a TA instrument model SDT-Q600.

The flowability level (spread) was measured through the set of flow table and circular truncated cone specified in ASTM C 230, but without the impacts of the flow table established by the standard procedure described in ASTM C 1437. The fresh stage air content and unit weight were determined through the standard procedure describer in ASTM C 185.

Prismatic mortar bars with a square section of one inch and a length of 30 cm, were casted and cured under standard conditions for 91 days, subsequently several 3 mm thick slices were cut form the specimens with a low-speed diamond saw, and prior their image analysis to perform quantitative energy-dispersive X-ray spectroscopy analysis (QEDS), the slices were immersed in isopropanol for 2 weeks to stop the hydration process. For the QEDS analysis, the samples were dried and mounted in resin, followed by polishing the surface and a later application of a 15 nanometers layer of carbon, in order to be analyzed with a Hitachi S-3400 N scanning electron microscope, for which the acceleration voltage and the working distance were set at 15 kV and 10 mm, respectively.

## 3. Experimental Results and Discussion

### 3.1. Compressive Strength

**Figure 2:**
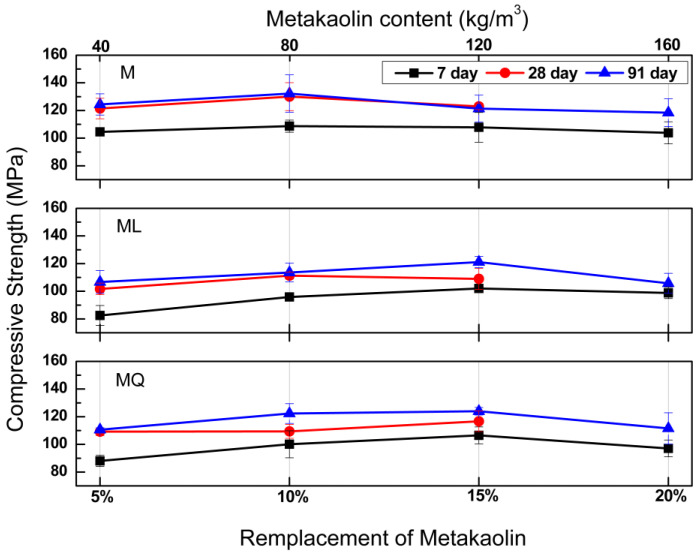
Compressive strength results at the ages of 7, 28 and 91 days for concretes with metakaolin (MK) and limestone (L) or quartz (Q) powders.

For the four concrete series evaluated in this work, compressive strength was determined for early, intermediate, and advanced ages to observe strength development at of ages of 7, 28 and 91 days, Figure 2 shows the compressive strength developments for mixtures MK, MKL and MKQ, as a function of the addition of metakaolin (5%, 10%, 25% and 20%). In practical applications, these concretes are very useful for the manufacture of precast elements, in which the strength at early ages is very useful to avoid damage to the elements during lifting and transport maneuvers, on the other hand, the strength at advanced ages is used in the structural design of concrete elements and structures.

In the upper part of Figure 2 it can be seen that for MK mixtures and for the three ages for which the compressive strength is reported, the additions of metakaolin greater than 5% do not modify the compressive strength, which suggests that in concretes with additions of 10%, 15% and 20%, the metakaolin is acting mainly as a filler. With compressive strengths of 109, 129 and 132 MPa at the ages of 7, 28 and 91 days, the best performance for MK mixtures was presented by the mixture with a metakaolin addition of 10%.

In this same Figure, we can see that for the four metakaolin additions (5%, 10%, 15% and 20%) and in relation to the results obtained for the MK mixtures, in the MKL and MKQ mixtures, the substitutions of 37.5% of the volume of cement by limestone or quartz powder, led for MKL mixtures to reductions of the compressive strength of 20.2, 11.9, 6.5 and 4.9, and 14.4, 14.4, 1.6 and 10.9 at the ages of 7 and 91 days respectively, as well as that for the MKQ mixtures with reductions of compressive strength of 15.4, 8.3, 1.9 and 5.8, and 11.2, 7.8, 0.8 and 5.9 at the ages of 7 and 91 days respectively. Based exclusively on the compressive strength, and on the results obtained at the age of 91 days, MKL and MKQ mixtures with 15% metakaolin, are the ones that contribute the most to reduce the ecological impact of the material, since with a replacement of 37.5% by volume of the cement by limestone or quartz powder, the strengths were practically equal to those corresponding to the MK mixture (1.6% reduction for the MKL mixture and 0.8% reduction for the MKQ mixture).

In comparison with the compressive strengths results of mixtures MK, it was expected that in mixtures MKL the potential reaction between metakaolin and limestone powder could generate similar or superior strengths as a result of the presence of carboaluminate hydration products [10], but the results obtained for mixtures MKQ suggest that in the MKL mixtures the reaction between metakaolin and limestone powder did not occur or that it was incipient, as well as that in low *w*/*c* systems the filler effect of limestone powder is more dominant.

It has been reported that the portlandite content of a given mixture, both in cases of limestone deficiency and excess, decreases with increasing metakaolin content, [18] although portlandite crystals have a marginal impact from mechanical point of view, so they do not provide any resistance to concrete, the best way to make this lime beneficial from a mechanical, durability and sustainability is to transform it into the so-called secondary C-S-H by reacting it with pozzolanic materials or slag [19], therefore the aforementioned, coupled with the reduction in the pozzolanic activity of MK between the ages of 28 and 91 days attributable to a high amount of kaolin, caused the reaction between limestone and metakaolin not to occur [20].

### 3.2. Bulk Electrical Resistivity (BER)

As an estimation of the potential concrete durability, cubic standard specimens were used to determine the BER, first according to the standard procedure described in ASTM C 1876 (Standard Method of Test for Surface Resistivity Indication of Concrete’s Ability to Resist Chloride Ion Penetration), and to the corresponding qualitative classification provided in this test method to evaluate also the probability of chloride ion penetration (High > 12, Moderate ≤ 12–21, Low ≤ 21–37, Very Low ≤ 37–254 and Negligible > 254 KΩ·cm), and second by correcting this limits according to the procedure proposed by W. Morris et al. in 1996 [21], leading to the following limits for the probability of chloride ion penetration in terms of BER (High > 6, Moderate ≤ 6–10.5, Low ≤ 10.5–18.75, Very Low ≤ 18.75–127 and Negligible > 127 KΩ·cm).

**Figure 3:**
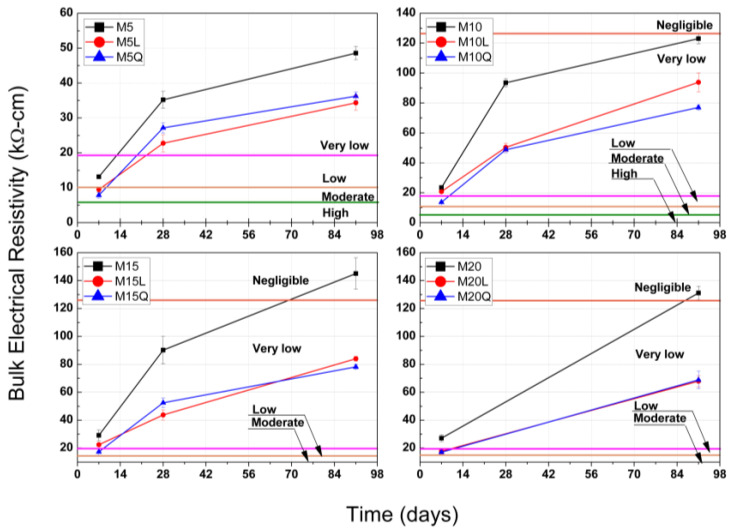
Bulk electrical resistivity results at ages between 7 and 91 days for concretes with metakaolin (M) and limestone (L) or quartz (Q) powders. A qualitative indication of the probability of chloride ion penetration, calculated for the BER results from those reported in AASHTO T358 is also included.

For mixtures MK5, Mk10, MK15 and MK20, the BER results reported in Figure 3, up to an age of 91 days, suggest that the additions of metakaolin provide the most beneficial matrix densification, for these mixtures the BER values at an age of 7 days were 13.5, 23.0, 30.0 and 28.0 kΩ.cm, respectively, corresponding to a low (10.5 ≤ 18.75 kΩ·cm) or very low (18.75 ≤ 127 kΩ·cm) probability of chloride ion penetration. For mixtures MK5, MK10 and MK15, the development of BER between the ages of 7 and 28 days were 1.05, 3.38 and 2.86 kΩ-cm/day, corresponding for this period to a net BER development of 45.8%, 58.2% and 41.7%, respective to the BER value obtained at 91 days, this performance indicate that the matrix densification is more significant in this period. The development of BER between the ages of 28 and 91 days was less significant with values of 0.20, 0.44 and 0.86 kΩ·cm/day, and contributes with 26.0%, 23.0% and 37.5% of the total BER obtained at 91 days. For mixtures MK5, MK10, MK15 and MK20 the BER results at 91 days were 48, 122, 144 and 130 kΩ·cm, corresponding to a very low (18.75 ≤ 127 kΩ·cm) or negligible probability of chloride ion penetration (>127 kΩ·cm). The abrupt and most significant level of densification that the mixtures MK5, MK10 and MK15 developed during the first 28 days was 74.0%, 77.0% and 62.5% (including the 28.1%, 18.9% and 20.8% developed the first 7 days, respectively) of the 100% developed at 91 days is attributed to the to the high reactivity of metakaolin. Even though mixture MK15 presented the highest BER at 91 days (144 kΩ·cm), mixture MK10 was the one that developed the highest level of densification during the first 28 days (77.0%).

The volume substitution of 37.5% of cement by either L or Q originated a significant BER reductions. At the ages of 7/28/91 days, the results of BER for mixtures M5L, M10L, M15L and M20 were lower than the ones presented by the correspondent mixtures M5, M10, M15 and M20 by 29.6/22.5/25.0%, 8.7/46.3/23.8%, 23.3/42.2/42.4% and 32.1/(n/a)/47.4% respectively, and at the same ages, the reductions for mixtures M5Q, M10Q, M15Q and M20Q were 40.7/35.2/29.2%, 43.5/47.3/36.9%, 40.0/51.1/45.8% and 35.7/(n/a)/48.5% respectively. The lower reactivity shown by the previous results is due to the significant reduction of the cement that also caused reductions in the development of BER between the ages of 7 and 28 days and between the ages of 28 and 91 days (7-28/28-91, in kΩ-cm/day), for mixtures M5L, M10L and M15L, the development of BER was 0.86/0.13, 1.40/0.67 and 1.38/0.49 kΩ-cm/day, and for mixtures M5Q, M10Q and M15Q the development of BER was 0.71/0.17, 1.74/0.44 and 1.24/0.54 kΩ-cm/day respectively. Although at the age of 7 days the mixtures with L or Q presents a moderate (6 ≤ 10.5 KΩ·cm) or low (10.5 ≤ 18.75 KΩ·cm) ability to resist chloride Ion Penetration, in the short term, all these mixtures improve their level of densification and present a very low (18.75 ≤ 127 KΩ·cm) permeability that will be improve as time passes, as illustrated by the development of BER for the periods of 7 to 28 days and 28 to 91 days. To compliment these results and, at the same time, to confirm if the contribution of L to the densification of the cementitious matrix was merely as a filler or if it reacts in the presence of metakaolin, Section 3.3, Section 3.4 and Section 3.5 present the results of the following microstructural analysis: thermogravimetric analysis, X-ray diffraction and X-ray spectroscopy.

### 3.3. Thermogravimetric Analysis

**Figure 4:**
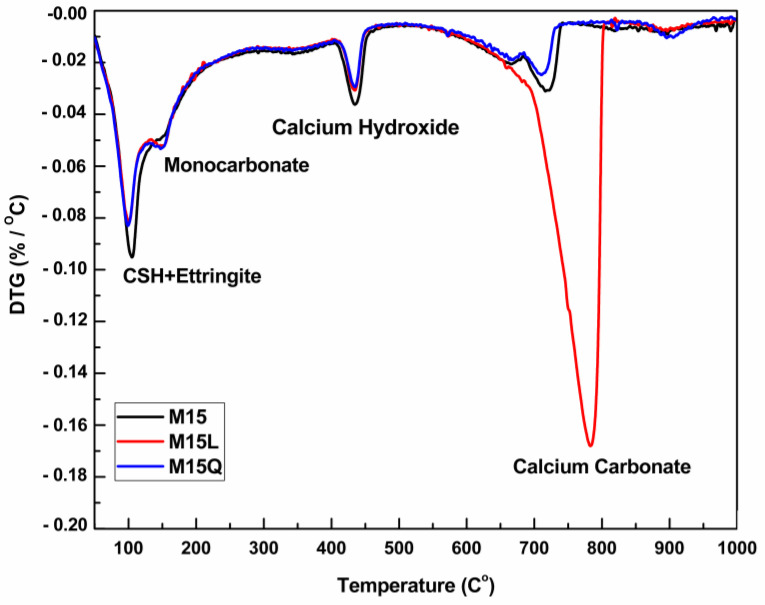
Derivative of the Thermogram for the paste fractions of mixtures M15, M15L and M15Q at 91 days.

The derivative thermogravimetric curves (DTG) for paste fractions of M15, M15L and M15Q appears in Figure 4 and evidence that the amount of calcium hydroxide and monocarbonate is almost the same, regardless of the substitution of cement by limestone powder. The presence of monocarbonate in the paste fraction of mixture M15Q is attributed to the presence of calcite in the cement. 

**Figure 5:**
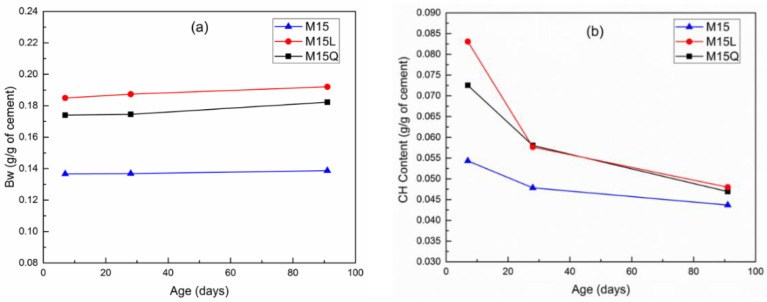
Development of bound water content (**a**) and calcium hydroxide content (**b**) between 7 and 91 days for mixtures M15, M15L and M15Q.

From the TGA analysis, the calcium hydroxide (CH) content was obtained through the tangent method, and the bound water (Bw) regarded as the mass between 35 °C and 1000 °C, minus the amount of carbon dioxide resulting from by the decarbonation of carbonate minerals. The changes of CH and Bw normalized to the amount of cement in the sample and in relation to time are summarized in Figure 5; this figure illustrates the progress in cement hydration and the reaction of metakaolin as the amount of CH decreases.

In Figure 5a, it can be observed that the substitution of cement by L or Q increases Bw by 4 and 3 g/g at an age of 7 days, and by 5 and 4 g/g at an age of 91 days [20], indicating a higher hydration level in mixtures M15L and M15Q, or that the replacement of cement by fillers significantly increases the hydration degree. On the other hand, results indicate that in mixture M the hydration level remained the same between 7 and 91 days. At a macro level, these results are in agreement with the compressive strength results reported in Figure 2. 

Regarding the reactivity of MK, Figure 5b shows that the pozzolanic reaction if this supplementary cementitious material mainly occurs between the ages of 7 and 28 days, since after 28 days the reaction remains mostly dormant. Even though it has been stated that in comparison with other fillers, in cementitious systems with limestone filler, the CH consumption increases as the limestone addition increases [4]. In our case, this assertion is not valid because the sample with quartz presented almost the same calcium hydroxide content between the ages of 28 and 91 days. The reduction in the pozzolanic activity of MK, between the ages of 28 and 91 days could be due to the fact that the MK used in this work has a high amount of kaolin [19].

As previously mentioned, and as can be seen in Figure 2, mixtures M15, M15L and M15Q presented similar compressive strength values, and are in agreement with the C-S-H contents presented in Figure 4 and in Table 4, with concentrations of C-S-H of 62%, 62% and 53.0% respectively, Being the C-S-H the hydration compound that have the highest influence in the compressive strength, despite the addition of Limestone or quartz fillers, the C-S-H content has remained similar [22].

Although the compressive strength values remained similar between mixtures M15, M15L and M15Q, a slight decrease in compressive strength was observed for mixture M15. As we can observe in Figure 5, this decrease is attributed to the lower amount of bound water in mixture M15, because the loss of bound water could significantly reduce the compressive strength, for which about 50% of the result depends, on the cohesive and bonding forces that occur in the C-S-H [23].

### 3.4. X-ray Diffraction

**Figure 6:**
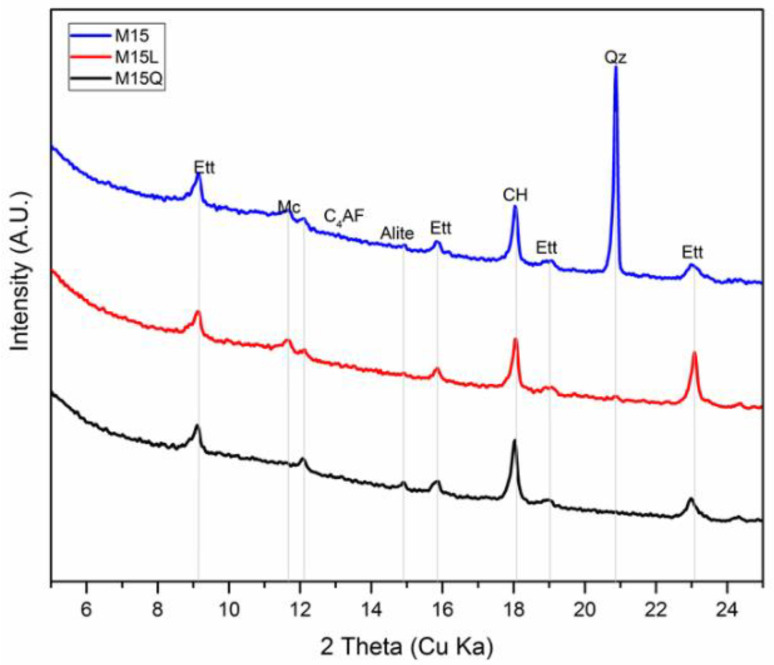
Mineral phases identified by XRD, in the paste fraction of mixtures M15, M15L and M15Q, after 91 days standard curing (ettringite: Ett, monocarbonate: Mc, tricalcium silicate: Alite, tretracalcium ferroaluminate: C4AF, calcium hydroxide: CH, and quartz: Qz).

In a Portland cement system with a filler, the hydration level will increase as the amount of bound water increases, and the phase dissolution can be monitored through an XRD analysis, to identify the mineral phases that are present in the sample, like the ones reported in Figure 6, for the paste fractions of mixtures M15, M15L and M15Q: ettringite, monocarbonate and calcium hydroxide. Unlike what has been reported in other works that used this technique to evaluate the hydration activity of cementitious systems consisting of Portland cement with limestone filler [10,24,25], in the samples evaluated in this work at 91 days the presence of the phase hemi carbonate or strätlingite was not found. 

To quantify the amount of the mineral phases reported in the difractograms presented in Figure 6, the information provided by the XRD difractograms was further analyzed trough the Xpert HighScore Plus 4.7a from PANalytical, and the Rietveld refinement method, including the determination of the background trough the bending factor functions of the software. In order to increase the robustness of the Rietveld refinement, a previous refinement of the anhydrous cement was performed to define the unit cell constants, and the profile variables that remained constant in the refinement of the hydrated samples [17]. Only the scale factor of the clinker phases was allowed to variate, and in the case of the hydration products, the unit cell was restricted to a variation of 1% and the profile parameter *w* was restricted to oscillate between 0.0001 to 0.2. Once the Rietveld refinement was finalized, the results were corrected by the Mass Attenuation Coefficient and normalized using the G factor method [26].

Additionally, to the mineral phases identified in Figure 6, the presence of the following phases was also detected and quantified through the Rietveld refinement method: belite (C2S), calcite, basanite, gypsum and amorphous content (CSH). The results of the refinement that appears in Table 4 confirm the very little amount of the monocarboaluminate phase, that was also identified by the TGA analysis. As reported previously from thermodinamical modelling and mass balance analysis [27], the results indicate that the reaction degree of limestone is minimum, still in conditions in which there is sufficient amount of aluminate, calcium and water, the production of such phase is scarce [25].

**Figure 7:**
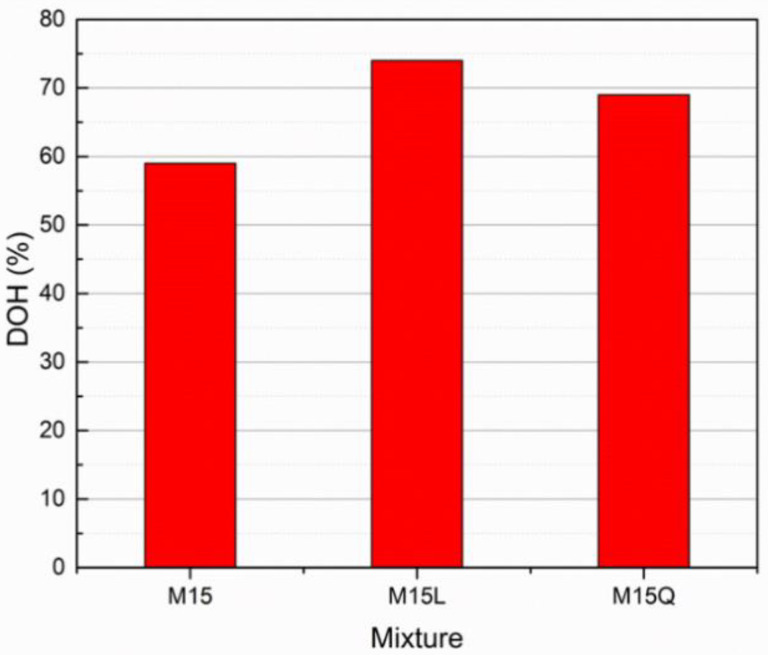
Cement’s Degree of Hydration at 91 days for paste fractions of samples M15, M15L and M15Q.

The degree of hydration was calculated according to Equation (1) with the difference in the concentration of the phases in the hydrated and unhydrated Portland cement (C_3_S, C_2_S, C_3_A and C_4_AF), the results are presented in the Figure 7.

(1) $$\mathrm{DOH}=\frac{{\left(C_{3}S,C_{2}S,C_{3}{A\;\mathrm{and}\;C}_{4}\mathrm{AF}\right)}_{\mathrm{anhydrous}}-{\left(C_{3}S,C_{2}S,C_{3}{A\;\mathrm{and}\;C}_{4}\mathrm{AF}\right)}_{\mathrm{sample}}}{{\left(C_{3}S,C_{2}S,C_{3}{A\;\mathrm{and}\;C}_{4}\mathrm{AF}\right)}_{\mathrm{anhydrous}}}$$

where:

DOH: Degree of hydration of the cement

(C_3_S, C_2_S, C_3_A and C_4_AF) anhydrous: Is the sum of clinker phases in the anhydrous cement.

(C_3_S, C_2_S, C_3_A and C_4_AF) sample: Is the sum of clinker phases in the hydrated sample.

Figure 7 clearly evidence the improvement in terms of hydration degree when fillers are used in a Portland cement based cementitious system, as the paste fractions for mixtures M15L and M15Q presented higher hydration degrees (74% and 69% respectively) in comparison with the one obtained for the paste fraction of mixture M15 (59%). From these results we can also highlight that in this comparison, the metakaolin addition and the low *w*/*c* ratio decreased the degree of hydration of the Portland cement, and that the substitution of 37.5% of the cement volume by L or Q fillers practically led to complete hydration of all cement. In this regard, when cements with a scarce amount of C3A are combined with metakaolin, they will present a low performance (decreased dissolution and hence low mechanical performance), attributed to a rapid decrease in the alkalinity of the pore solution, which is not present in systems made exclusively with Portland cement [24]. However, due to rheological and workability issues, in UHPC mixtures it is always recommended to use cements with low amounts of aluminates and to define the most compatible polycarboxylate admixture (structure and dosage) [28].

Finally, an indirect measurement of the pozzolanic reaction was performed by determining the amount of calcium hydroxide consumed in the paste. For this purpose, first the Ca/Si ratio of the CSH was determined through Equation (2) as a function of the Alite dissolution and of the calcium hydroxide production, on paste fractions without metakaolin and with *w*/*c* of 0.25 and 0.4, that previously to the analysis remain under standard cured for 91 days [29]. The Ca/Si ratios obtained for pastes with *w*/*c* of 0.25 and 0.4 were 1.85 and 2.01, respectively.

(2) $$\frac{\mathrm{Ca}}{\mathrm{Si}}=3-\frac{{\%\mathrm{CH}\;*\;\%C}_{3}S}{{\mathrm{mwCH}\;*\;\mathrm{mwC}}_{3}S}$$

where:

Ca/Si: Is the calcium to silica ratio of the C-S-H that results from the reaction of Alite

%CH: Is the weight in percentage of calcium hydroxide normalized for the cement content in the sample.

mwCH: Is the molar weight of calcium hydroxide. 

%C_3_S: Is the weight in percentage of the C_3_S that reacts and normalized for the cement content in the sample.

mwC_3_S: Is the molar weight of the C_3_S. 

This Ca/Si ratios were used to estimate trough Equations (3) and (4), the amount of calcium hydroxide produced in the reaction

(3) $${\%\mathrm{CH}}_{0.25}={\%C}_{3}S\times \frac{\mathrm{mwCH}}{{\mathrm{mwC}}_{3}\;S}\times \left(1.15\right)$$

(4) $${\%\mathrm{CH}}_{0.40}={\%C}_{3}S\times \frac{\mathrm{mwCH}}{{\mathrm{mwC}}_{3}\;S}\times \left(0.99\right)$$

where:

%CH_0.25_ = Is the estimated percentage of calcium hydroxide resulting from the hydration of the Alite in the cement, for a paste fraction with a *w*/*c* of 0.25 analyzed after 91 days of standard curing. 

%CH_0.4_ = Is the estimated percentage of calcium hydroxide resulting from the hydration of the Alite in the cement, for a paste fraction with a *w*/*c* of 0.40 analyzed after 91 days of standard curing.

Finally, the consumed CH was obtained by the following equation.

(5) $${\%\mathrm{CH}}_{c}=\frac{{\%\mathrm{CH}}_{0.40\mathrm{or}\;0.25}-\%\mathrm{CH}}{{\%\mathrm{CH}}_{0.40\;\mathrm{or}\;0.25}}$$

where

%CHc: Is the estimated consumed CH 

%CH_0.4 or 0.25_ = As described before, is the estimated CH production. The result obtained for paste with a *w*/*c* of 0.25 were used to calculate the 91-day theoretical CH content for mixture M15, and the result obtained for a paste with a *w*/*c* of 0.40 were used to calculate the 91-day theoretical CH contents for mixtures M15L and M15Q.

**Figure 8:**
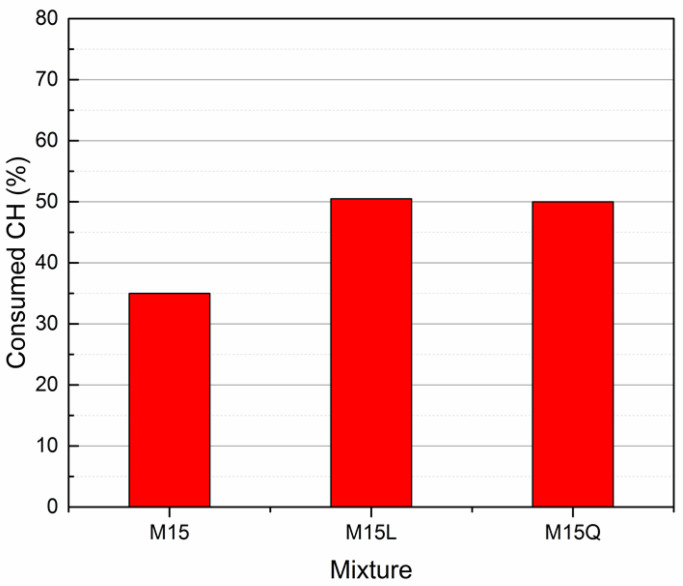
Consumed Calcium Hydroxide in the paste fraction of mixtures M15, M15L and M15Q after 91 days of standard curing.

In order to be able to compare the consumed CH in the three mixtures, the value was normalized dividing the consumed CH by the 91-day theoretical CH, to obtain a percentage, which is presented in the Figure 8. Results indicate that with the cement replacement by L or Q, the portlandite consumption increases 15%. The almost equal performance presented by mixtures M15, M15L and M15 Q in terms of compressive strength is attributed to the increase of both, the hydration degree and the portlandite consumption. 

### 3.5. X-ray Spectroscopy

In a binary Portland cement-metakaolin based cementitious system, one of the reaction products will be the Calcium-aluminum-silicate-hydrates (C-A-S-H). To determine the composition of this hydrate, bars of UHPC with dimensions of one square inch and a length of 10 inches were casted and standard cured for 91 days. From these specimens, slices of 3 mm thick were cut with a diamond saw and stored immersed in isopropanol for two weeks to remove as much free water as possible. Then the samples were dried by the solvent exchange method, and after they were mounted in resin, polished, and coated with a 15 nm carbon layer. Finally, the samples were introduced to a scanning electronic microscope (SEM) equipped capable to perform Quantitative Energy-Dispersive X-ray Microanalysis (QEDS).

**Figure 9:**
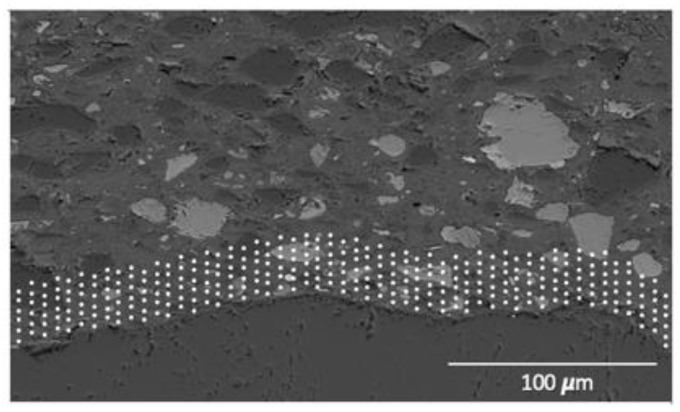
Example of the spot analysis on the paste to define the C-A-S-H composition.

To perform quantitative measurements in the cement paste, standards were used for every relevant element within the cement paste: C_2_S for Ca and Si, C_3_A for Al, olivine for Mg and Fe, anhydrite for S, orthoclase for K, tugtupite for Na and Cl, and sphene for Ti. The obtained spectra were post-processed with the NIST software DTSA-II, that allow us to obtain quantitative data from the information of previously measured standards with a known chemical composition. The Duane-Hunt limit was used to exclude points where local charging could have taken place. The investigation was performed in the paste around the aggregates in layers of 3 μm, for a total thickness of 50 μm within the paste around the aggregate particle (Figure 9).

**Figure 10:**
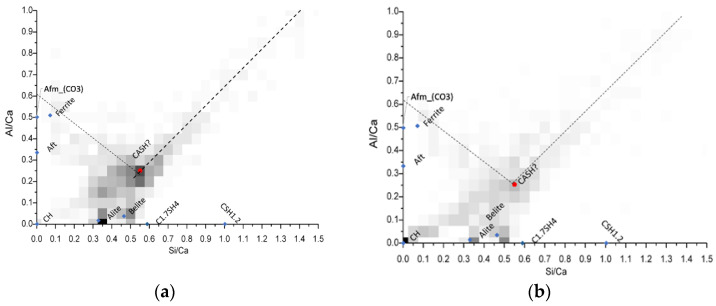

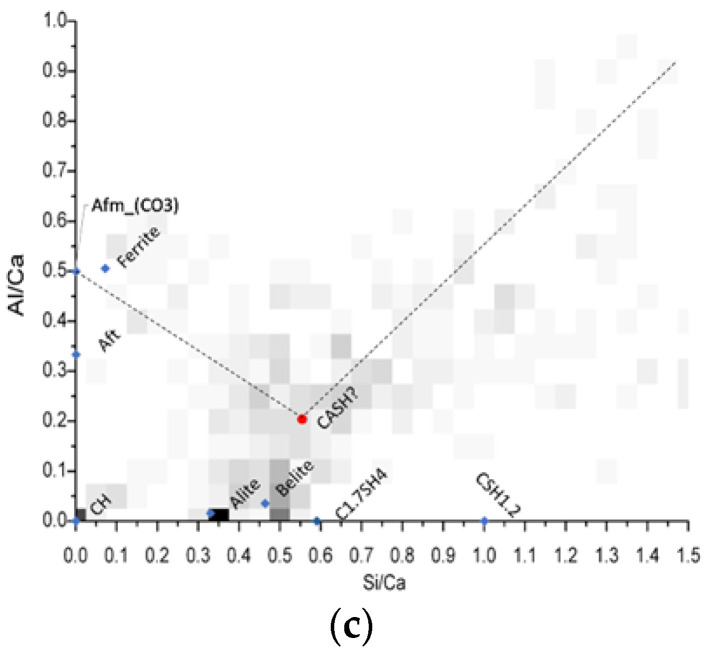
Bivariate Histograms for the definition of C-A-S-H composition for mixtures M15 (**a**) M15L (**b**) and M15Q (**c**) after 91 days of standard curing.

For this purpose, bivariate histograms were constructed with the Si/Ca vs Al/Ca molar ratios (Figure 10). The red dots in the intersection are representative of the C-A-S-H composition, and its average elemental ratios are summarized in Table 5. The different molar ratios reported in Table 5 shows very similar C-A-S-H compositions among the mixtures evaluated through this analysis, and also that in comparison with the typical Al/Ca ratio for ordinary Portland cement (0.05) [30], the ternary Portland cement-filler-metakaolin cementitious systems and the binary cement-metakaolin system presented AL/Ca ratios of 0.20 or higher. It is noted the great aluminum incorporation in the C-A-S-H passing from the 0.05 Al/Ca ratio in normal OPC pastes to more than 0.2. This heavy Al incorporation results from the high kaolinite content in our calcined clay [31]. Wrapping up the results, we could see that the limestone filler is not specially more beneficial when used in conjunction with calcined clays in low water/cement environments, further evaluation with other type of fillers must be done to expand the metakaolin based ternary systems to not only calcite. Nevertheless, the use of any kind of fillers enhances the hydration degree in systems with very low water availability. 

### 3.6. Comparison in Cost and Environmental Impact

Table 6 present a cost and CO_2_ emissions for the paste fractions of a conventional UHPC with silica fume and the optimized mix with metakaolin and limestone filler reported in this work. Cost used to estimate the values presented in Table 6 was considering the cost by 1 MT of ordinary Portland Cement (OPC), Silica Fume (SF), and Metakaoilin (MK) and this cost were 116 USD, 260 USD, and 250 USD, respectively [32,33].

In the other hand, the CO_2_ emissions for each powder aforementioned, were obtained through research in several references, and the values obtained were 930 kg-CO_2_/tons, 8 kg-CO_2_/tons, 393 kg-CO_2_/tons by OPC, SF and MK respectively [34,35,36].

**Table 6:** Comparison of cost and CO_2_ emission attributed to the fine powder fraction in UHPC’s with SF or MK and L.

Material	UHPC with SF			UHPC with MK		
	Proportions (MT/m^3^)	Cost (USD/m^3^)	CO_2_ Emissions (kg-CO_2_/m^3^)	Proportions (MT/m^3^)	Cost (USD/m^3^)	CO_2_ Emissions (kg-CO_2_/m^3^)
OPC	0.79 [36]	91.64	734.7	0.50	58	465
SF	0.20 [36]	52.00	1.6	-------	-------	-------
MK	------	-------	-------	0.12	30	47.16
L	------	-------	-------	0.25	1.25	0
Total	0.99	143.64	736.3	0.87	89.25	512.16

The cost of an UHPC is highly influences by the cost of the fibers Table 6 present a cost and CO_2_ emissions for the paste fractions of a conventional UHPC with silica fume and the optimized mix with metakaolin and limestone filler reported in this work.

According to the results presented in Table 6, and to the references from which the costs of the materials were obtained, the powder fraction of the optimized UHPC with MK and limestone filler was 38.0% cheaper than the UHPC with SF. Regarding the emissions attributable exclusively to the ingredients that constitute the powder fraction, the optimized mixture presents a reduction of 30.4% of CO_2_ emissions.

## 4. Conclusions

Based on the results and commentaries of this work, the following conclusions can be drawn:

1. In terms of compressive strength and sustainability, the UHPC that presented the best performance was the one with a metakaolin addition of up to 15% by mass of cement of metakaolin and with a substitution of 37.5% of the cement volume, by either limestone or quartz filler. Higher additions of metakaolin are ineffective.
2. As a durability index, the bulk electrical resistivity indicates that metakaolin addi-tions between 10% and 20% by mass of the cement, significantly improves the ability of these concretes to resist chloride ion penetration and to reach the highest level of imperviousness considered in AASHTO T358 (negligible for BER > 127 KΩ·cm), since an age of 70 days or later.
3. Results of TGA revealed that the hydration of both, cement and metakaolin, is very active between 7 and 28 days, and dormant at later ages, as well as that the substitu-tion of cement by limestone or quartz fillers (37.5% by volume), improves the hydra-tion reaction and as a result of the significant cement reduction that also decreased the amount of portlandite by 15%.
4. XRD results indicate that the low water-cement ratio and the high reactivity of me-takaolin didn’t allow the formation of monocarboaluminate.
5. From the QEDS it was possible to quantify the increased aluminum incorporation at 15% MK, passing from a normal C-A-S-H Al/Ca ratio of 0.05 to 0.20. Both quartz and limestone filler replaced samples yield to the same type of C-A-S-H.

In terms of compressive strength and bulk electrical resistivity the mixture with a metakaolin addition of 15% and a limestone or quartz substitution of 37.5% of the cement volume is identified as the optimum UHPC mixture, because a higher addition of metakaolin didn’t improve these properties. As a durability index, the bulk electrical resistivity indicates that metakaolin additions between 10% and 20% by mass of the cement, significantly improves the ability of these concretes to resist chloride ion penetration and to reach the highest level of imperviousness considered in AASHTO T358 (negligible for BER > 127 KΩ·cm), since an age of 70 days or later.

The hydration of the binary cement (HS + MK) evaluated in this work exhibit the most significant activity between the ages of 7 and 28 days, and after, hydration continue at a very low rate up to an age of 91 days. The presence of either, limestone or quartz filler (35% by volume of the cement) when added to the binary cement, improves the hydration reaction, and the significant reduction of cement led to a portlandite reduction of 15%.

The low water-cement ratio and the highly reactive metakaolin didn’t allow the formation of monocarboaluminate, and the aluminum incorporation resulting from the dosage of metakaolin (15%) increased the Al/Ca content in the C-A-S-H hydrated phase. Both fillers led to the formation of the same type of phase C-A-S-H hydrated phase.

In terms of cost and carbon footprint (CO_2_ emissions), the optimized mixture with metakaolin and limestone filler led to a reduction of 38.0% and 30.4% respectively.

## Figures and Tables

**Table 1:** Physical and chemical properties for HS, MK, Q and L, obtained by X-ray fluorescence, pycnometry, air permeability (Blaine), BET tests, and laser granulometry.

Oxide Amount or Property (Weight, %)	Cementitious Materials		Powders	
	HS	MK	Q	LS
SiO_2_	21.8	52.1	99.60	5.10
TiO_2_	0.18	1.60	0.01	0.04
Al_2_O_3_	4.00	44.4	0.10	0.80
Fe_2_O_3_	3.14	0.40	0.02	0.27
Mn_2_O_3_	0.038	-----	0.002	0.008
MgO	1.90	0.10	0.10	1.30
CaO	63.10	-----	-----	51.20
Na_2_O	0.05	0.10	-----	-----
K_2_O	0.56	0.10	-----	0.34
P_2_O_5_	0.09	-----	-----	0.01
SO_3_	2.37	-----	-----	0.06
SrO	0.10	-----	-----	0.03
ZnO	0.02	-----	-----	-----
Loss of ignition	2.60	1.0	0.10	40.90
Equivalent alkali (Na_2_Oeq)	0.42	0.20	-----	0.22
Density, g/cm^3^	3.18	2.59	2.75	2.71
Blaine surface, m^2^/kg	438.00	-----	-----	-----
BET surface, m^2^/g	-----	10.66	-----	-----
Mean particle size (d50), μm	14.00	4.10	19.00	17.00

**Table 2:** Normalized chemical phase composition for HS, MK, Q and L.

Chemical Phase (wt %)	HS	MK	L
C_3_S	52.0	-----	-----
C_2_S	18.0	-----	-----
C_3_A cubic	1.0	-----	-----
C_3_A orthorhombic	2.0	-----	-----
Ferrite	10	-----	-----
Periclase	1.0	-----	-----
Gypsum	1.0	-----	-----
Bassanite	3.0	-----	-----
Calcite	5.0	-----	75.0
Quartz	-----	-----	3.0
Anatase	-----	2.0	-----
Dolomite	-----	-----	3.0
Amorphous	7.0	98.0	19.0

**Table 3:** Proportions and fresh stage properties for the mixtures evaluated in this work.

Material, Parameter or Property	Mixtures											
	M5	M5L	M5Q	M10	M10L	M10Q	M15	M15L	M15Q	M20	M20L	M20Q
*w*/*c* weight ratio	0.25	0.40	0.40	0.25	0.40	0.40	0.25	0.40	0.40	0.25	0.40	0.40
MK by weight of cement	0.05	0.08	0.08	0.1	0.16	0.16	0.15	0.24	0.24	0.2	0.32	0.32
Water (kg/m^3^)	199	199	200	199	199	200	199	199	200	200	200	200
HS (kg/m^3^)	797	499	500	797	499	500	798	499	500	800	500	500
L (kg/m^3^)	---	250	---	---	250	---	---	251	---	---	256	---
Q (kg/m^3^)	---	---	245	---	---	245	---	-	245	---	---	259
MK (kg/m^3^)	40	40	40	80	80	80	120	120	120	160	160	160
Solids in HRWRA (kg/m^3^)	12	9	9	12	9	9	12	9	9	14	11	11
Sand (kg/m^3^)	1369	1377	1381	1328	1336	1339	1287	1296	1298	1239	1246	1246
Unit weight (kg/m^3^)	2436	2407	2399	2421	2390	2397	2424	2380	2378	2352	2330	2318
Air content before vibration (%)	1.0	1.0	1.0	2.0	1.0	1.0	2.0	2.0	2.0	3.0	2.0	3.0
Air content after vibration (%)	0.0	0.0	0.0	0.0	0.0	0.0	0.0	0.0	0.0	2.0	0.0	0.0
Mini cone Spread without vibration (cm)	30	32	33	28	31	33	27	30	32	24	28	27

**Table 4:** Phase distribution obtained through the Rietveld refinement method for the paste fractions of mixtures M15, M15L and M15Q, after 91 days of standard curing.

Phase	Mixtures		
	M15	M15L	M15Q
C3S	15.0%	6.0%	7.0%
C2S	6.0%	3.0%	4.0%
C4AF	3.0%	2.0%	2.0%
Calcite	3.0%	20.0%	1.0%
Calcium Hydroxide	5.0%	3.0%	3.0%
Ettringite	4.0%	3.0%	3.0%
Quartz	-----	-----	25.0%
Bassanite	-----	1.0%	-----
Gypsum	1.0%	1.0%	1.0%
Monocarbonate	0.00%	0.10%	0.20%
Amorphous content/CSH	62.0%	62.0%	53.0%

**Table 5:** C-A-S-H composition for mixtures M15, M15L and M15Q after 91 days of standard curing.

Mixture	Ca/Si		Al/Ca		S/Ca		Fe/Ca	
M15	1.82	±0.05	0.25	±0.01	0.06	±0.01	0.03	±0.02
M15L	1.82	±0.05	0.25	±0.02	0.04	±0.02	0.03	±0.03
M15Q	1.80	±0.05	0.20	±0.04	0.03	±0.01	0.04	±0.06
